# Application of the Indian Academy of Cytologists’ Recommendations for Reporting Serous Fluid Cytology

**DOI:** 10.7759/cureus.81787

**Published:** 2025-04-06

**Authors:** Hariom Meena, Nikita Singh, Arjun Singh, Anand Bhadkariya, Nidhi Rai

**Affiliations:** 1 Department of Pathology, Government Medical College & Hospital, Datia, Datia, IND; 2 Department of Pathology, Government Medical College Satna, Satna, IND

**Keywords:** effusion, immunocytochemistry, indian academy of cytologists (iac), malignant cells, risk of malignancy

## Abstract

Background: Cytological evaluation is often the first line of investigation in the clinical setting of serous effusions. Reporting according to risk stratification can aid in a better understanding between clinicians and pathologists and the triage of patients.

Materials and methods: All serous effusion samples were collected and reported according to the guidelines of the Indian Academy of Cytologists. The risk of malignancy for each category was assessed, and immunological typing for atypical cases was performed.

Results: A total of 364 fluid samples from different age groups ranging from nine to 82 years were examined, of which 206/364 (56.5%) were pleural fluid and 158/364 (43.5%) were peritoneal fluid samples. The majority of fluids belonged to category II in both pleural (137/206, 66.5%) and peritoneal fluids (113/137, 71.5%), whereas only 9/206 (3.5%) of pleural fluid and 8/133 (5.1%) of peritoneal fluid samples were malignant. The maximum risk of malignancy was assessed for category V, followed by categories IV, III, I, and II. Immunocytochemical analysis showed cytokeratin (CK) positivity in two pleural fluid samples, whereas carcinoembryonic antigen (CEA) positivity was observed in seven pleural and eight peritoneal fluid samples.

Conclusion: The Indian Academy of Cytologists' recommendations for reporting effusion cytology utilize a five-tiered reporting system that is feasible and mitigates subjective variation in reporting.

## Introduction

Cytology for serous effusions is a common investigation encountered in pathology laboratories. Clinicians usually send serous fluids to the laboratory to diagnose whether effusion is due to benign or malignant processes. An imbalance between the generation and reabsorption of serous fluid leads to serous effusions in the peritoneal and pleural cavities [1,2]. Their existence is always regarded as pathological, and they are indicative of both benign and malignant disorders. Serous fluid cytology is a cost-effective technique that aids in the diagnosis, staging, and origin of malignancy.

The Indian Academy of Cytologists (IAC) released guidelines for the collection, processing, interpretation, and reporting of serous effusion fluid samples at the beginning of 2020. The goals of these guidelines are to ensure good cytopathology practice, achieve uniformity across all laboratories, and implement a standard reporting format with consistent recommendations in similar contexts. The cases were classified into one of the five suggested categories following cytological evaluation. The IAC recommends five categories: category I, unsatisfactory for evaluation; category II, no malignant cells detected/benign cellular changes; category III, atypical cells, not otherwise specified; category IV, atypical cells, suspicious for malignancy; category V, malignant cells seen [3,4]. Immunocytochemical analysis of malignant cells present in the serous fluid can play a significant role in identifying the primary site of origin.

The present study aimed to categorize all serous effusions according to IAC reporting guidelines for serous fluid and immunocytochemical typing of malignant cells in serous effusions.

## Materials and methods

The present study is a retrospective analytical study, which was conducted on 364 effusion fluid samples (ascitic fluid and pleural fluids) for a duration of six months. All samples were collected from different wards to ensure that they met the requisition form with some necessary details such as name, age, gender, hospital registration number, clinical diagnosis with related history, and date and time of collection. We also correlated radiological and endoscopic findings with previous diagnosis and treatment, wherever available. We ensured that the collected fluid was transported as soon as possible and processed all fluids within two hours of collection.

Inclusion and exclusion criteria

We have included samples of all age groups from zero to above 60 years. We have included both genders in our study. All the samples for which proper history and clinical details were available were included.

Exclusion criteria included samples for which proper history and follow-up were not available and patients who were positive for HIV infection.

Physical examination was performed, and the following points were taken into consideration: volume, color, appearance, turbidity, and clot formation.

Routine cytology examination

The fluid sample was collected in a test tube and centrifuged at 3000 rpm for 10 minutes. The supernatant was separated, and the sediment at the bottom was used for smear preparation. Five slides were prepared, air-dried, and fixed with methanol on one slide for routine cytological examination and the rest for immunocytochemical examination. May-Grunwald Giemsa (MGG) staining was used for cytological evaluation. After cytological evaluation, we categorized the fluids according to the IAC into categories I, II, III, IV, and V. Further immunocytochemical examination was performed in cases that belonged to categories III, IV, and V on cytological examination. The risk of malignancy (ROM) for each category was also calculated in cases where follow-up data were available from histopathology, radiology, or clinical records. Since the present study is a simple analytical study, no complicated calculations or statistics are required.

Immunocytochemical examination

Immunocytochemical examination (ICC) was performed using the polymeric (EnVision FLEX Mini Kit, Dako, Agilent, Santa Clara, CA) technique of cell smear preparation, which was prepared as previously described. These were air-dried and kept at 2-8℃ till further use. The EnVision system is based on dextran polymer technology, which permits the binding of a large number of enzyme molecules to a secondary antibody via a dextran background. Prediluted and ready-to-use antibodies from Dako were used.

## Results

A total of 364 fluid samples from different age groups, ranging from nine to 82 years, were examined. Of the 364 fluid samples collected, 206 (56.5%) were pleural fluids and 158 (43.5%) were peritoneal fluids. Of the 206 pleural fluid samples, 133 (66.5%) were of males and 73 (36.5%) were of females, whereas out of the total 158 peritoneal fluid samples, 86 (54.4%) were of females and 72 (45.6%) were of males.

**Figure 1:**
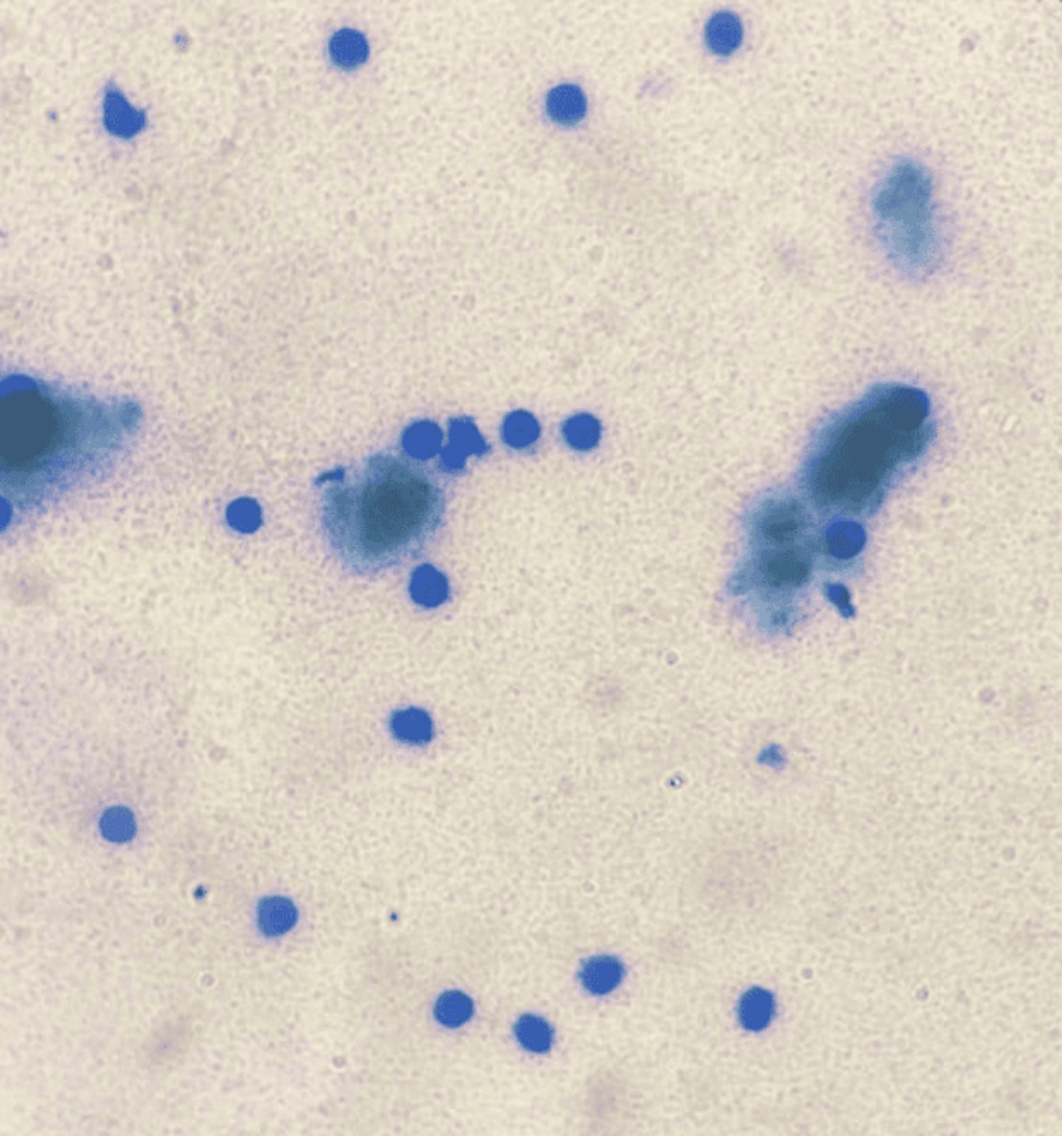
Smears of cases belonging to category II (benign) reported as inflammatory effusions showing scattered lymphocytes against a proteinaceous background.

**Figure 2:**
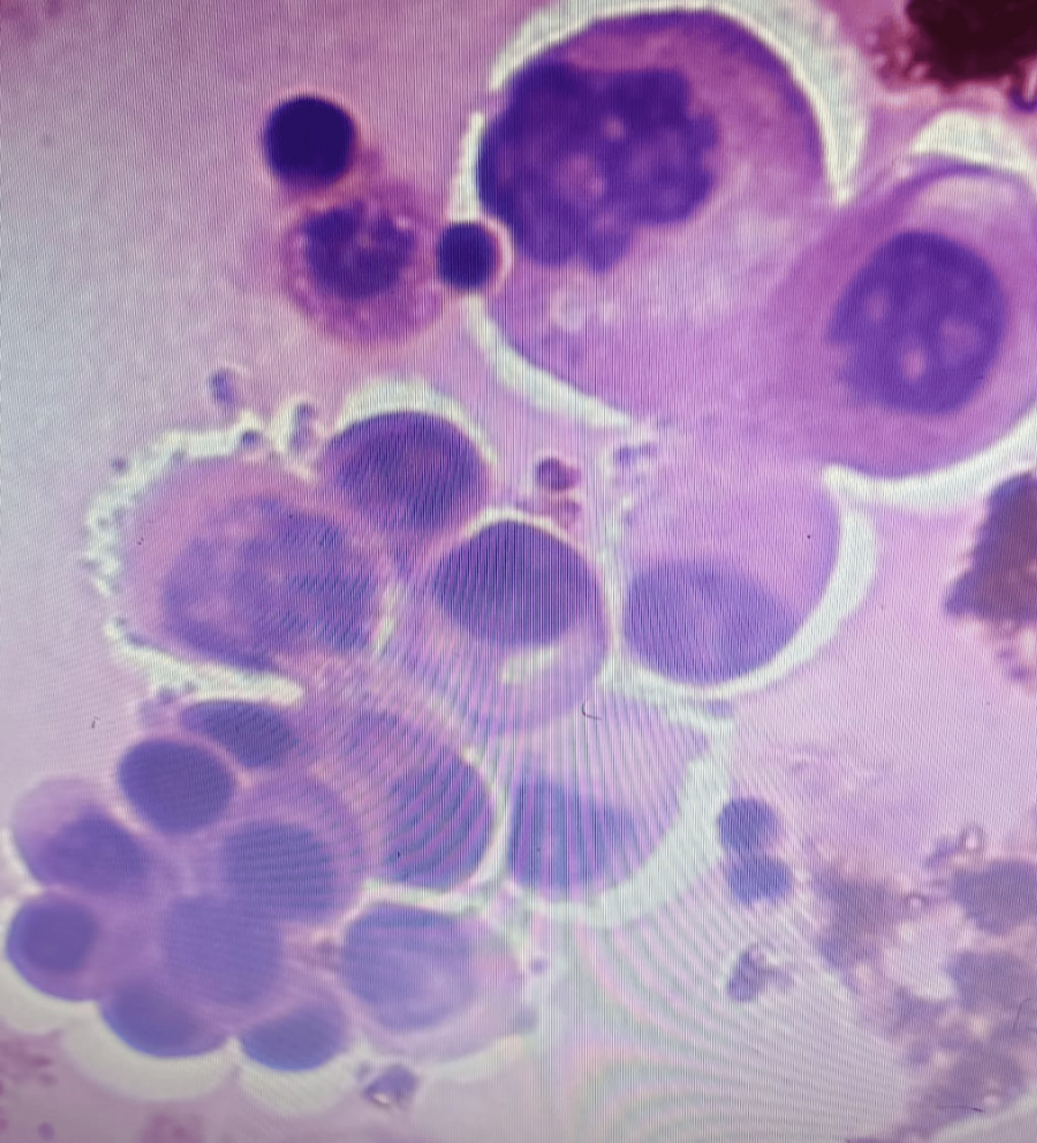
Smear of a case belonging to category V, that is malignant. Malignant cells with a high nuclear-to-cytoplasmic ratio, irregular nuclear membrane, and a moderate amount of cytoplasm.

As shown in Table 1, out of 206 pleural effusion samples, 19/206 (9.2%) belonged to category I, 137/206 (66.5%) belonged to category II (Figure 1), 27/206 (13.1%) were of category III, 14/206 (6.7%) were of category IV, and 9/206 (3.5%) were from category V (Figure 2). Of the 158 peritoneal fluid samples, 12/158 (7.6%), 113/158 (71.5%), 18/158 (11.3%), 7/158 (4.4%), and 8/158 (5.1%) belonged to categories I, II, III, IV, and V, respectively.

**Table 1:** Distribution of studied fluid samples according to the IAC categories. IAC: Indian Academy of Cytologists.

Category	Pleural fluid		Peritoneal fluid	
I	19	9.2%	12	7.6%
II	137	66.5%	113	71.5%
III	27	13.1%	18	11.3%
IV	14	6.7%	07	4.4%
V	09	3.5%	08	5.1%

The risk of malignancy was calculated after the follow-up of patients. Clinical diagnosis, radiological findings, and smear findings were correlated, and the final diagnosis was made on follow-up. Of the 364 cases, 135 were available for follow-up. Table 2 shows the risk of malignancy, which was 20% for category I, 17.9% for category II, 46.6% for category III, 75% for category IV, and 100% for category V.

**Table 2:** Risk of malignancy in IAC categories. IAC: Indian Academy of Cytologists; ROM: risk of malignancy.

	Category I	Category II	Category III	Category IV	Category V
Benign	10	67	15	8	00
Malignant	2	12	7	6	8
Total	12	79	22	14	8
ROM	20%	17.9%	46.6%	75%	100%

**Figure 3:**
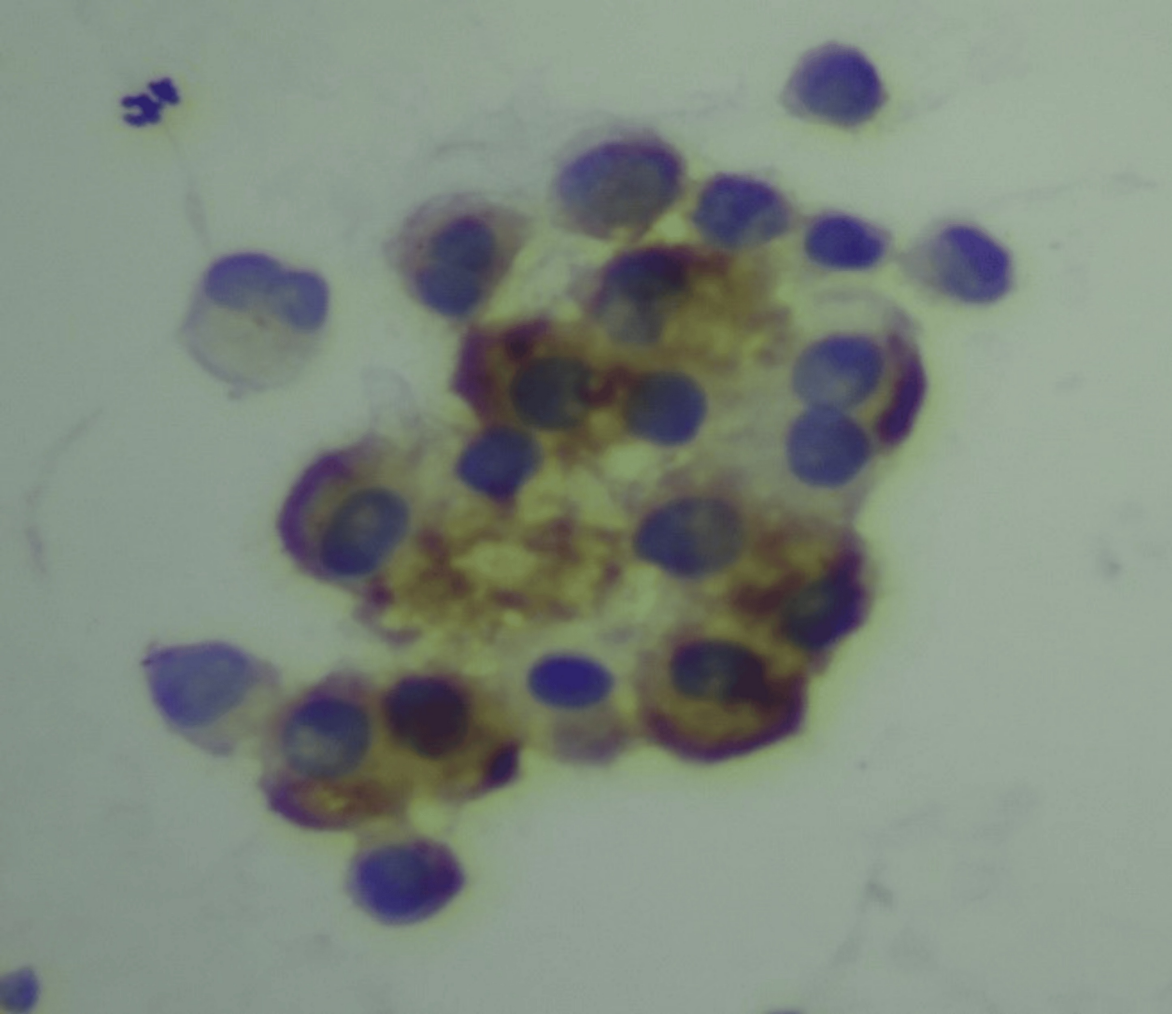
Immunocytochemistry for CEA showing positivity of CEA in malignant effusion. CEA: carcinoembryonic antigen.

**Figure 4:**
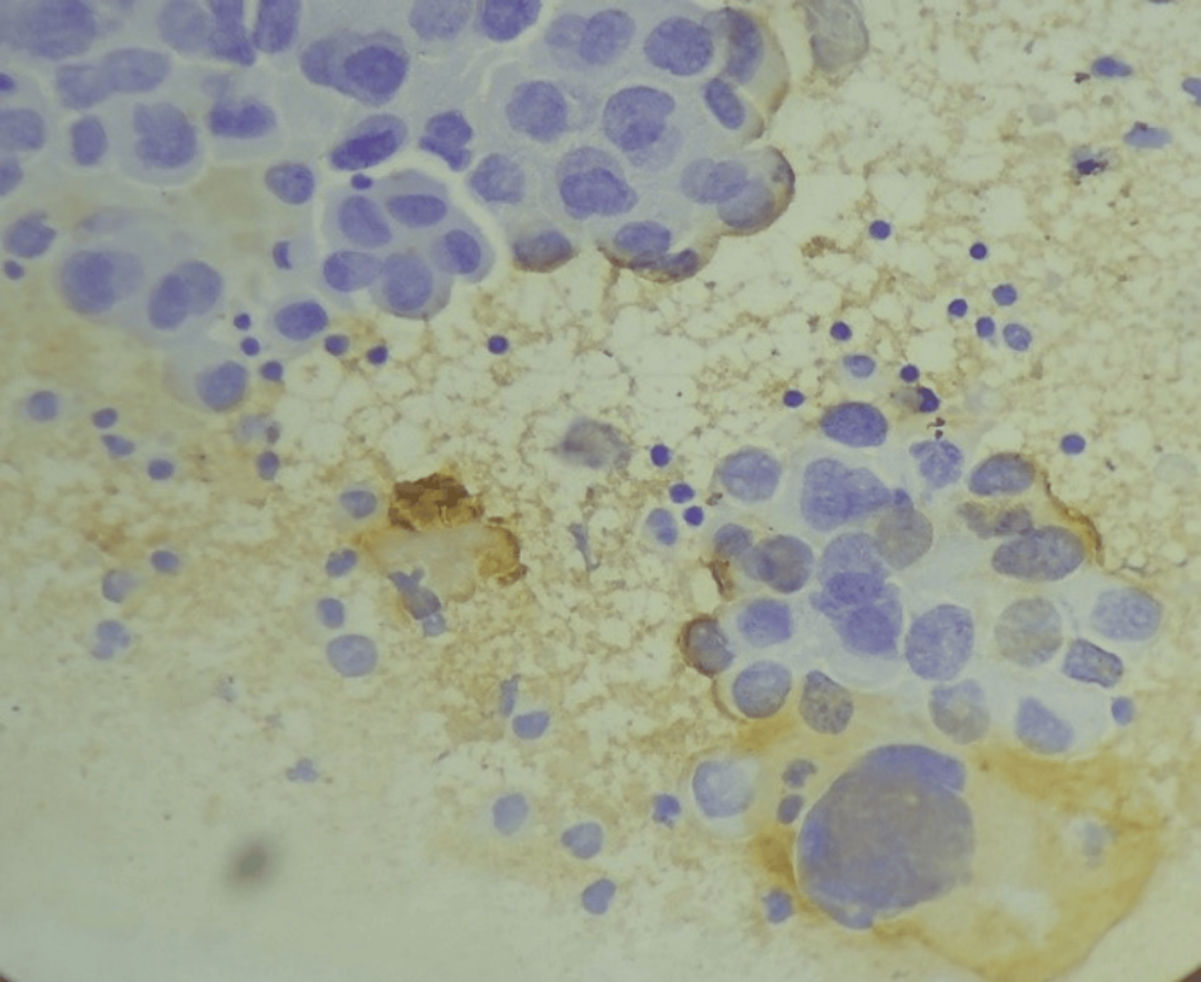
Immunocytochemistry for CK showing cytoplasmic positivity for CK. CK: cytokeratin.

We applied immunocytochemistry to fluids belonging to categories III, IV, and V (Table 3). Of the 50 pleural fluid smears, seven were positive for carcinoembryonic antigen (CEA) (Figure 3) and two smears showed positivity for cytokeratin (CK) (Figure 4). However, out of 33 ascitic fluid samples, eight showed positivity for CEA, and CK remained negative for all.

**Table 3:** Immunocytochemical analysis of fluids in IAC category III, IV, and V. IAC: Indian Academy of Cytologists; CEA: carcinoembryonic antigen; CK: cytokeratin.

Effusions	CEA		CK		Total
	Positive	Negative	Positive	Negative	
Pleural fluid	07	43	02	31	50
Ascitic fluid	08	42	00	33	33

## Discussion

Daily cytopathology practice includes cytological assessment of body cavity fluids as a fundamental component. Cytological examination of effusion smears is rapid and cost-effective and can be used efficiently in outpatients as well as in admitted patients. In the current investigation, effusion samples were categorized into five diagnostic groups according to the IAC guidelines [4]. Reporting serous fluids according to the IAC guidelines provides a basis for standardizing the processing and diagnostic categories and improving communication.

In the present study, 21/364 (5.7%) effusion samples were classified as category I (nondiagnostic). These samples were either too little (less than 10 milliliters), contaminated with bacteria, improperly anticoagulated and clotted, or contained too much anticoagulant, which caused crystals to develop. Repeat evaluation was advised in all these cases, along with proper instructions for sample collection and transportation [4]. Similar results were observed in a study performed by Jha et al. [5] in which 41/961 (4.26%) cases fell into category I. Studies conducted by Kundu et al. [6] and Kalita et al. [7] showed 35/1340 (2.6%) and 5/342 (1.46%) of the cases in category I, respectively.

In the present study, most cases (250/364, 71.2%) were observed in category II (Figure 1), i.e., benign. Similar results were observed by Kundu et al. [6], Jha et al. [5], and Kalita et al. [7]. In the present study, 45/364 (12.36%) cases represented category III, that is atypical, which is comparatively more than other studies such as Kundu et al. [6] (17/1340, 1.3%), Jha et al. [5] (5, 5.2%), and Kalita et al. [7] (9/342, 2.63%). This difference in results may be due to inter-observer errors, over-reporting of some cases, or technical faults.

In the present study, 21/364 (5.76%) cases were observed in category IV, which is similar to the results of studies conducted by Kundu et al. [6] (59/1340, 4.4%), Jha et al. [5] (31/961, 3.25%), and Kalita et al. [7] (20/342, 5.84%). We found that 17/364 (4.67%) cases belonged to the malignant category (Figure 2), which is similar to the study conducted by Kalita et al. [7] (20/342, 5.84%) and different from the studies conducted by Jha et al. [5] and Kundu et al. [6] in which 79/961 (8.22%) and 275/1340 (20.5%) of cases were malignant, respectively (Table 4).

**Table 4:** Comparison of the distribution of cases in various categories.

Study and year	Non-diagnostic	Benign	Atypical	Suspicious for malignancy	Malignant
Kundu et al. [6] (2021)	2.6%	71.2%	1.3%	4.4%	20.5%
Jha et al. [5] (2022)	4.26%	83.7%	5.2%	3.25%	8.22%
Kalita et al. [7] (2023)	1.46%	84.2%	2.63%	5.84%	5.84%
Present study (2024)	8.51%	68.68%	12.36%	5.76%	4.67%

In present study, we interpret that ROM for cases belonging to category I is 20%, which is similar to studies conducted by Kundu et al. [6] and Jha et al. [5] and different from the study conducted by Kalita et al. [7] in which they found 0% ROM for category I of the IAC. The ROM for category II was 17.9%, which is similar to the results of Kundu et al. [6] and Jha et al. [5]. In the present study, the ROM for category III was found to be 46.6%, which is similar to the results of studies conducted by Kundu et al. [6]​​​​​​​ and Kalita et al. [7]. The ROM for category IV in the present study was 75%, while it was 50% in the study conducted by Kalita et al. [7], 90% in the study conducted by Jha et al. [5], and 94.4% in a study by Kundu et al. [6]. The ROM for category V was 100%, which was similar in all of the above studies (Table 5).

**Table 5:** Comparison of ROM in various categories. ROM: risk of malignancy.

Study and year	Category I	Category II	Category III	Category IV	Category V
Kundu et al. [6] (2021)	20%	16.7%	50%	94.4%	100%
Jha et al. [5] (2022)	21.42%	14.9%	33.3%	90%	96.4%
Kalita et al. [7] (2023)	0%	4.4%	50%	50%	100%
Present study	20%	17.9%	46.6%	75%	100%

In a study conducted by Nance et al. [8], the authors found that CEA shows positivity in 20/27 (77%) malignant cases and CK shows positivity in 25/27 (93%) cases of malignant effusion. However, in the present study, we performed immunocytochemistry with CEA in 50 pleural and 33 ascitic fluid samples and found that it was positive (Figure 3) in 7/50 (14%) pleural and 8/33 (24%) ascitic fluid samples. The results of the present study vary from those of a study conducted by Nance et al. [8]​​​​​​​ because they only performed ICC on malignant cases, whereas we performed ICC on fluids belonging to categories III, IV, and V. Lee et al. [9] found that CEA was positive in 25 (89%) out of 28 malignant cases, and 24 (86%) out of 28 cases were epithelial membrane antigen (EMA) positive; however, in present study, we did not use EMA. In the present study, we found that eight out of 33 cases (24%) showed positivity for CK (Figure 3).

The limitation of the present study is that it is a single-institute study, and the sample size is not very high. During the study, some of the patients did not come for proper follow-up, and we did not study the samples of HIV-positive patients.

## Conclusions

Effusion cytology is an important diagnostic tool for evaluating benign and malignant fluids. An accurate cytological diagnosis is ensured by standardizing the reporting terminologies with little interobserver variation, which is necessary for appropriate patient management. These standardized reporting methods can aid in improving a report's comprehension. This reporting system can be easily applied to effusion fluids for better patient management and effective communication with clinicians.
